# Vehicle-Type Detection Based on Compressed Sensing and Deep Learning in Vehicular Networks

**DOI:** 10.3390/s18124500

**Published:** 2018-12-19

**Authors:** Yinghua Li, Bin Song, Xu Kang, Xiaojiang Du, Mohsen Guizani

**Affiliations:** 1 State Key Laboratory of Integrated Services Networks, Xidian University, Xi’an 710071, China; liyh@stu.xidian.edu.cn (Y.L.); xkang0591@gmail.com (X.K.); 2 Department of Computer and Information Sciences, Temple University, Philadelphia, PA 19122, USA; dxj@ieee.org; 3 Department of Electrical and Computer Engineering, University of Idaho, Moscow, ID 83844, USA; mguizani@ieee.org

**Keywords:** vehicle classification, target detection, compressed sensing, convolutional neural network, saliency map

## Abstract

Throughout the past decade, vehicular networks have attracted a great deal of interest in various fields. The increasing number of vehicles has led to challenges in traffic regulation. Vehicle-type detection is an important research topic that has found various applications in numerous fields. Its main purpose is to extract the different features of vehicles from videos or pictures captured by traffic surveillance so as to identify the types of vehicles, and then provide reference information for traffic monitoring and control. In this paper, we propose a step-forward vehicle-detection and -classification method using a saliency map and the convolutional neural-network (CNN) technique. Specifically, compressed-sensing (CS) theory is applied to generate the saliency map to label the vehicles in an image, and the CNN scheme is then used to classify them. We applied the concept of the saliency map to search the image for target vehicles: this step is based on the use of the saliency map to minimize redundant areas. CS was used to measure the image of interest and obtain its saliency in the measurement domain. Because the data in the measurement domain are much smaller than those in the pixel domain, saliency maps can be generated at a low computation cost and faster speed. Then, based on the saliency map, we identified the target vehicles and classified them into different types using the CNN. The experimental results show that our method is able to speed up the window-calibrating stages of CNN-based image classification. Moreover, our proposed method has better overall performance in vehicle-type detection compared with other methods. It has very broad prospects for practical applications in vehicular networks.

## 1. Introduction

Throughout the past decade, vehicular networks have captured a great deal of interest in both the industrial and academic fields. The increasing number of vehicles in traffic has led to challenges in traffic regulation. In the 1970s, only a magnetic coil could be used to detect vehicle types; now, radar, ultrasonic, and infrared images, and surveillance videos, are widely used for detection [1]. With an increasing number of digital video-surveillance devices widely deployed on traffic roads and vehicles, vehicle vision-detection methods have become an actively researched topic in recent years. Vehicles offer a perfect platform for urban sensing applications, as they can be equipped with a variety of sensing devices that may continuously monitor the environment around the traveling vehicles. Vehicle-type detection is also an important research topic with diverse applications in intelligent transportation systems, driverless vehicles, and road safety. Its main purpose is to extract the different features of vehicles from videos or pictures captured by traffic surveillance so as to identify the types of vehicles and then provide reference information for traffic monitoring and control.

In this paper, we focus on the detection and classification of vehicles in realistic traffic pictures. Traditionally, such methods consisted of two parts. First, some regions are selected in an image and labeled as the targets; the sliding-window method is generally used for this. Next, features are extracted from these regions. Then, robust classification methods are applied to determine their types. These classification methods include decision trees, support vector machines (SVM), and neural networks [2]. There exist two major difficulties in traditional detection methods. Conventional area selection is based on window sliding, which involves high computational complexity and results in enormous redundant regions. Furthermore, in traditional image processing, the features of an image are extracted from the pixel domain, so the scale of the feature extracting network is quite large if the image has many pixels. So, target detection via conventional machine learning methods has encountered bottlenecks.

In 2006, Hinton et al. put forward the concept of deep learning [3] that brought researchers’ ideas into a new realm. The advent and development of deep learning have had a significant impact on detection and classification methods. Deep learning is largely based on the principle of neurons in the human brain. Relying on big data, it progressively extracts the high-dimensional information of images using convolution and layer-by-layer sampling. In this way, objects are classified more accurately and purposefully.

Here, our purpose is to propose a step-forward vehicle detection and classification method that applies a saliency map and convolutional neural network (CNN). Specifically, we used compressed-sensing (CS) theory to generate a saliency map to label the vehicles in an image, which a CNN scheme then classifies. CS [4,5,6] is applied to reduce the dimensionality of the input data and simultaneously extract the internal features of images from the projections using singular value decomposition (SVD) for producing the optimal projection matrix. Many existing methods need to scan the whole image for the target label during the detection step, and most of these scans are redundant and avoidable. We introduced the concept of a saliency map, on the basis of which the image is searched for target vehicles. The use of the saliency map in this step minimizes redundant areas. At present, there exist many methods to generate saliency maps. We used CS to measure the image of interest and obtain the saliency in the measurement domain. Because the data in the measurement domain are much smaller than that in the pixel domain, we can generate saliency maps at a low computation cost and a faster speed. Then, based on the saliency map, the target vehicles are identified and then classified into different types using the CNN.

The remainder of this paper is constructed as follows. Section 2 lists some relevant work. In Section 3, we briefly introduce the correlative theoretical basis, including CS and CNN. Then, the proposed method based on CS and CNN is described in detail in Section 4. The explanation, illustration, and analysis of the experimental results are given in Section 5. Finally, a summary of this paper is presented in Section 6.

## 2. Related Work

The initial deep-learning method recognizes objects well, but it does not have the sliding-window function. In order to improve upon the weaknesses in deep learning, a large number of models and improved algorithms have sprung up. Improved CNNs, such as RCNN [7], SPP [8], and Faster RCNN [9], can quickly perform window calibration and recognize objects in various degrees. It can be said that object detection has entered a new stage. Conventional feature-extraction methods rely on prior knowledge, while CNN has a certain invariance to geometric transformation, deformation, and illumination. It can effectively overcome the variability in vehicle appearance and adapt to training data [10]. The deep-learning model based on CNN has been one of the most popular methods in the fields of target detection [11,12] and image classification [13,14,15]. Several papers (e.g., References [16,17,18,19]) have studied related issues.

Recently, CNN has obtained excellent results in many challenging classification tasks. A considerable number of studies have been carried out on this topic in recent years. The authors in Reference [20] used deep neural networks (DNN) and viewed the detection procedure as a regression problem. In another work [15], AlexNet and sliding windows were used to produce a method of image localization using CNN to detect and classify the images. In 2014, Ross et al. proposed an RCNN scheme that combined a region proposal network and CNN to replace the sliding window [21]. It was a major breakthrough in target detection using deep learning. The method in References [8,22] was realized by the combination of conventional machine learning and deep learning. Then, many optimized methods were developed, such as Fast RCNN [23], Faster RCNN [9], YOLO [24], and SSD [25].

Applying a Haarr-like feature pool and incremental learning method AdaBoost, Wen et al. proposed a rapid deep-learning method for vehicle classification [26]. Shen et al. presented a novel CS-based CNN model to classify images [2], using CS at the input layer to reduce consumed time. A fine-grained vehicle-classification method based on deep learning was presented in Reference [27]. In Reference [10], Li applied the Faster RCNN model to traffic scenarios and utilized self-built MIT [28] and Caltech [29] vehicle data as the test data, which improved the average target accuracy and detection rate. However, these aforementioned methods extract the features and detect the objects in the pixel domain. They fail to exploit the inherent relationship between the image characteristics in the pixel domain and those in the measurement domain. In this paper, we establish this relationship, which is the basis for generating the saliency map. Then, the saliency map is used to label the target region. Finally, a CS-based CNN accomplishes vehicle classification. The experimental results demonstrate that the method achieves a high accuracy for detecting main types of vehicles, namely, cars, minibuses, trucks, and SUVs.

## 3. Background Theory

### 3.1. Compressed Sensing

For completeness, we briefly introduce the fundamental background of CS. The emergence of CS has tremendously affected signal acquisition and signal recovery [4,5,6], because signal compressibility or sparsity is of great significance. Suppose that *x* is a discrete signal with size *n*; if it has no more than *r* nonzero values, then *x* is called “*r*-sparse”. A signal may have no sparsity in some domains. Fortunately, we can always find a certain domain where signal *x* can be considered sparse with an appropriate basis.

Based on CS theory, if a signal can be sparsely represented, the signal can be recovered. In fact, most natural images can be sparsely represented under a specific basis, so we can compress the original image. Denoting the sparse representation basis as $\Psi =[\psi_{1},\psi_{2},\psi_{3}\cdots \psi_{N}]$, the signal can be represented as:

(1) $$x=\sum_{j=1}^{n}\psi_{j}\theta_{N}=\Psi \theta$$

where $\theta_{i}$ is a representation coefficient *x* on the basis Ψ. Generally, if *x* can be sparsely represented under the basis Ψ, then Ψ is the sparse basis.

Choosing a random matrix as the measurement matrix, the measurement process can be formulated as:

(2) y=Φx=ΦΨθ=Pθ

where P=ΦΨ is a projection matrix with an M×N size; *M* and *N* are the numbers of the rows and the columns of the projection matrix, respectively. Then, *y* is termed as the measurement vector. The components of *y* are the measurements.

If a signal is analyzed or processed using measurements, sparse representation coefficients, and the sparse basis, then we can refer to it as signal processing in a measurement domain.

To reconstruct the sparse coefficients, the projection measurement matrix should satisfy the (restricted isometry property (RIP):

(3) $$1-\varepsilon \leq \frac{{∥P\nu ∥}_{2}}{{∥\nu ∥}_{2}}\leq 1+\varepsilon$$

where ε>0, and *v* is an arbitrary *k*-sparse vector. In general, the signal can be perfectly recovered if measurement matrix Φ and sparse basis Ψ are uncorrelated.

### 3.2. CNN

The CNN is a high-precision classification algorithm that has been developed in recent years. Especially in the field of image recognition, CNN has almost replaced the traditional method of image-feature recognition. With the further development of computer hardware, such as the GPU, the CNN has emerged from the laboratory and entered all aspects of people’s lives. From a functional point of view, the classical CNN structure can be divided into two parts, feature extraction and feature mapping. On the whole, we can still regard the CNN as a classifier. In detail, the output of each layer in the CNN structure can be regarded as another expression of data. Based on this feature, the image can be further manipulated and processed. At present, the key to the perfect operation of CNN and its variants are large-scale training data, which we often call Big Data.

**Figure 1:**
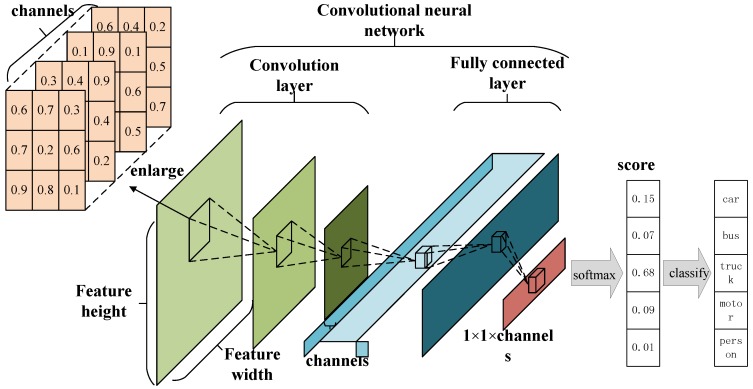
Diagram of the vehicle-type detection method based on compressed sensing (CS) and deep learning.

As shown in Figure 1, CNNs take the original image as input and generate corresponding feature maps as the output. The number of network layers directly influence the effectiveness of data processing, and this argument has empirical value: processing ability is reduced if the number of layers is set to be small; on the other hand, if the number is large, the whole network is too complicated. So, in this work, we employed five convolutional layer nodes, where each node is in the form of a stack followed by a MaxPooling layer. The structure of the CNN is in Figure 1. In this structure, each convolutional layer adopts a small 3×3 region as a receptive field with the step of one pixel. So, a convolutional stack that contains three convolutional layers has a receptive field of 7×7 with a reduction in network parameters.

## 4. Proposed Method

In this study, we addressed vehicle-type detection problems using saliency maps and deep learning. We focused on three issues. First, we built the relationship between an image’s frequency domain and measurement domain based on CS. Second, the saliency map was generated in the measurement domain for labeling the target regions, that is, the target detection process. Then, we used the CS-based CNN model to complete the vehicle classification.

### 4.1. Correlation between the CS Measurement and Frequency Domains

In this study, we extracted the saliency map of an image by analyzing measurements obtained via CS theory in the measurement domain, where the saliency map is used to demonstrate the salient features in the pixel domain. Traditional methods often generate a saliency map by analyzing the mass data in the pixel and frequency domains. Therefore, we built a linear relationship between an image’s frequency and measurement domains based on CS to show the reasonability and feasibility of the proposed method.

From Section 3.1, we know that the signal-measurement process can be formulated as:

(4) $$\begin{array}{c} y_{j}=\Phi x_{j}\left(j=1,2,\cdots ,N\right)\\ x_{i}=\Psi \theta_{i},y_{i}=\Phi \Psi \theta_{i}=P\theta_{i} \end{array}$$

where P=ΦΨ is the projection matrix, $y_{i}$ is the measurement vector, and Ψ is the sparse representation basis. Covariance can be calculated by:

(5) $$C_{y}=\frac{1}{m}\left[y_{1},y_{2},\cdots ,y_{m}\right]{\left[y_{1},y_{2},\cdots ,y_{m}\right]}^{T}-y_{0}y_{0}^{T}$$

At the same time, we can easily obtain mean value and variance as E(P)=0,D(P)=1/m. So,

(6) $$\sum_{i=1}^{m}\sum_{j=1}^{n}P_{ij}∼N\left(0,n\right)$$

and

(7) $$\frac{1}{m}\left(P_{1}+P_{2}+\cdots +P_{m}\right)\approx \vec{0}\Rightarrow y_{0}y_{0}^{T}=\vec{0}$$

where *m* and *n* represent sample numbers in the measurement domain and frequency domain, respectively.

Thus,

(8) $$\begin{array}{c} C_{y}\approx \frac{1}{m}\left[Y_{1},Y_{2},\cdots ,Y_{m}\right]{\left[Y_{1},Y_{2},\cdots ,Y_{m}\right]}^{T}\\ \;=\frac{1}{m}\left[\begin{array}{cccc} P_{1}\theta_{1} & P_{2}\theta_{1} & \cdots & P_{m}\theta_{1}\\ P_{1}\theta_{2} & P_{2}\theta_{2} & \cdots & P_{m}\theta_{2}\\ \vdots & \vdots & & \vdots \\ P_{1}\theta_{k} & P_{2}\theta_{k} & \cdots & P_{m}\theta_{k} \end{array}\right]\left[\begin{array}{cccc} P_{1}\theta_{1} & P_{1}\theta_{2} & \cdots & P_{1}\theta_{k}\\ P_{2}\theta_{1} & P_{2}\theta_{2} & \cdots & P_{2}\theta_{k}\\ \vdots & \vdots & & \vdots \\ P_{m}\theta_{1} & P_{m}\theta_{2} & \cdots & P_{m}\theta_{k} \end{array}\right]\\ \;=\frac{1}{m}{\left\{\left[\begin{array}{c} P_{1}\\ P_{2}\\ \vdots \\ P_{3} \end{array}\right]\left[\theta_{1},\theta_{2},\cdots ,\theta_{k}\right]\right\}}^{T}\left\{\left[\begin{array}{c} P_{1}\\ P_{2}\\ \vdots \\ P_{3} \end{array}\right]\left[\theta_{1},\theta_{2},\cdots ,\theta_{k}\right]\right\}\\ \;=\frac{1}{m}{\left(P\theta^{T}\right)}^{T}\left(P\theta^{T}\right)=\frac{1}{m}\theta P^{T}P\theta^{T} \end{array}$$

Then, the final covariance matrix can be obtained, represented as:

(9) $$C_{y}\approx \frac{1}{m}\theta P^{T}P\theta^{T}\approx \frac{1}{m}\theta \theta^{T}$$

while

(10) $$C_{\theta }\left(j,i\right)=E\left[\theta_{j}\theta_{i}\right]-E\left[\theta_{j}\right]E\left[\theta_{i}\right]$$

Therefore, we can conclude that the following linear relationship exists:

(11) $$C_{\theta }\approx \frac{m}{n}C_{y}$$

In our previous work [30], we theoretically proved, in detail, the approximately linear relationship between the cross-covariance matrices in the measurement domain and frequency domain. The images in the frequency domain and pixel domain are also closely related. Based on this result, we can more efficiently analyze the salient features of an image in the measurement domain by using far fewer measurements than we would in the pixel domain.

### 4.2. Saliency Map in the Measurement Domain

A saliency map is essentially a feature map which extracts the salient regions from the original image according to the saliency of the image, usually in black and white. The so-called salient area refers to the area that has a low correlation with the surrounding area. Generally, it is the “prominent” part of the image. From a subjective perspective, the salient region is the main part of the image: that is, it is the image region that people are most interested in. Therefore, the choice of salient regions is often very subjective. Saliency maps are widely used in image segmentation and other fields.

**Figure 2:**
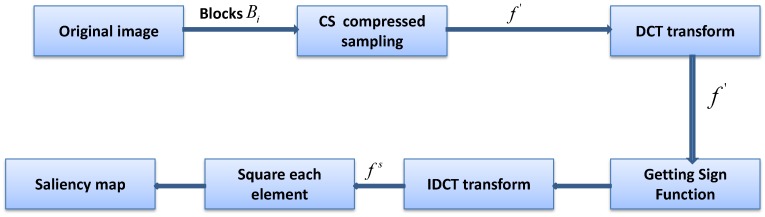
Diagram of extracting a saliency map in the measurement domain based on CS theory.

There exist various ways to generate saliency maps directly through the pixel domain. However, the more accurate the saliency map generation algorithm, the longer the time consumed. This is because the generation of the saliency map necessitates the analysis of every pixel of the original image. The more complex the algorithm and the clearer the image, the greater the number and complexity of the pixels to be processed. CS is an excellent dimension reduction tool. In our previous work [31], we proposed a method to obtain a trained dictionary directly by using video data measurements and then keeping the sparse components and generating a saliency map. However, this saliency map is simply generated by analyzing the sparse distribution of different regions without consideration of the specific content of one region, and its goal is to present the significant degree of a frame in the video data. Such a saliency map is not precise enough. In the present study, we generated an accurate saliency map using the method shown in Figure 2, based on which the window calibration of the suspected objects can be performed efficiently. The original image can be measured by means of CS. The size of the measured matrix will be much smaller than that of the original image. Analyzing the saliency region of the original image through the measured value matrix can greatly shorten the detection time.

An image can be divided into redundant and significant regions. If the image is sparsely represented, the sparse coefficients with values far from zero can be restored to salient regions, while the coefficients with values near zero correspond to redundant regions. In principle, a region is significantly different from the surrounding area, which means that there are obvious pixel differences between the region and the surrounding area. Reflected in the frequency domain, it is equivalent to the region which completes a jump from a low frequency to a high frequency. Because of this jump, the correlation between the frequency domain inside and outside of the region must be very small. We derived a positive correlation between the image’s measurement domain and frequency domain in the above section. So, our goal was to find the low-correlation part in the measurement domain which corresponds to the salient region of the original image. Next, we introduce the saliency detection algorithm, which is used to extract the salient regions from the measurement domain. The process diagram is as follows.

As shown in Figure 2, the specific saliency-extraction steps in the measurement domain are as follows:

- The original image is divided into sub-blocks of the same size, denoted as $B_{i},i=1,2,\cdots ,n$, which do not overlap with each other. All sub-blocks are measured by the same measurement matrix with original sampling rate $M_{0}$. Then, the measurement-value matrix is obtained.
- A discrete cosine transform for *f* is used to obtain compressed measured value matrix $f^{\prime }$.
- The sign function is obtained for each element of compressed measurement matrix $f^{\prime }$.
- The discrete cosine inverse transform of compressed matrix $f^{\prime }$ of the signed function is carried out to obtain saliency matrix $f^{s}$.
- Square enhancement matrix $f^{se}$ is obtained to apply the square operation to every element in the saliency analysis matrix $f^{s}$.
- The average value of the *i*th row in $f^{se}$ is compared with a threshold of 1: if the value is greater than 1, sub-block $B_{i}$ is significant, and the block is colored black; otherwise, the block is nonsignificant and the block is colored white. Thus, the saliency map is obtained.

### 4.3. Saliency Map-Based Window Calibration

Of the whole process of target detection and classification, the window calibration of suspected objects is the precondition of accurate target detection. The disadvantage of the traditional sliding-window method is that a large number of redundant areas cannot be eliminated, which results in numerous invalid calibration and recognition instances. The core of the selective search method is the design of a similarity computation strategy. A single strategy is easy to incorrectly merge, so the similarity calculation is very complex. This becomes the bottleneck of the selective search method.

**Figure 3:**
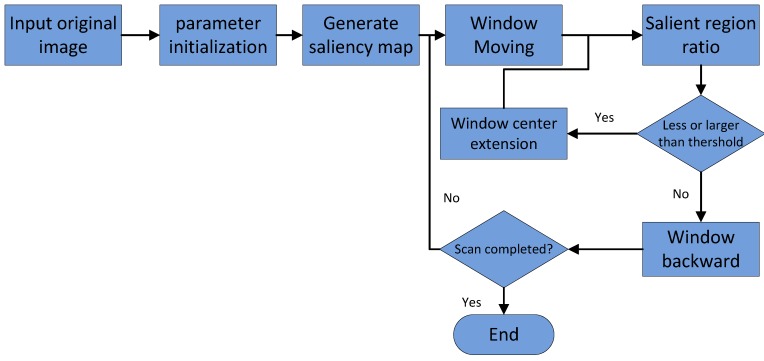
Saliency map-based window calibration.

In Section 4.2, we introduced the saliency-region detection method in the measurement domain. The redundancy region of the saliency map using our method was much smaller than that of traditional pixel-domain methods. The salient blocks generated in our proposal are colored only black and white to retain the main structure of the image, as it eliminates any interference caused by color and pixel texture changes. We can acquire window calibrations by following the steps shown in Figure 3.

### 4.4. Classification Using CNN

**Figure 4:**
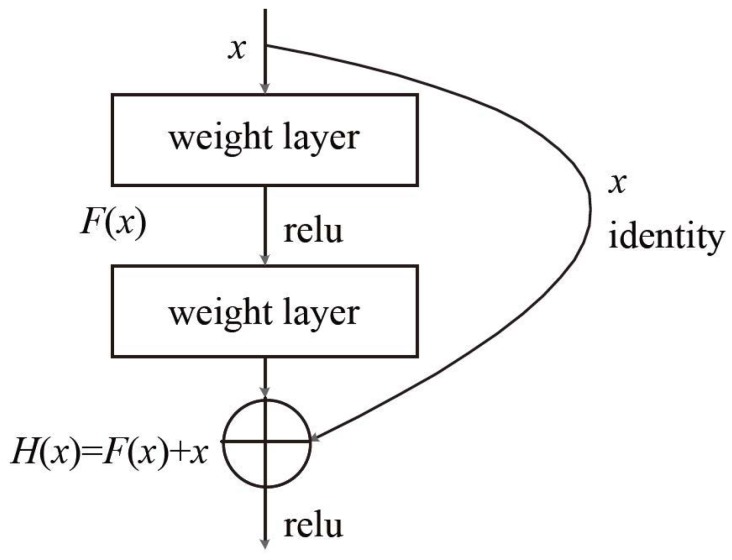
Residual network unit with the addition of a fast connection.

Deep-learning-based classification methods unify the feature extraction and classifier functions into one model. Feature extraction is automatically learned based on a large number of training data. The ResNet model proposed by [32] in 2015 has greatly improved the classification accuracy of pictures. The structure of ResNet is shown in Table 1. Here, we adopted the ResNet50 model. The main advantage of ResNet is that it can use a deeper network to solve the problem of increased training error with increased network layers. To solve this problem, the traditional plain network structure has been adjusted in ResNet. The key to the structure of ResNet is the addition of a quick identity link to the basic network unit (shown in Figure 4). Here, relu is the abbreviation of Rectified Linear Units, namely the activation function of a neuron in CNN, H(x) is the ideal map, F(x) is the residual map, and H(x)=F(x)+x. By transforming the objective fitting function H(x) into the fitting residual function F(x), the output is transformed into the superposition of the fitting value and the input, which makes the network more sensitive to the small fluctuations between output H(x) and input *X*.

**Figure 5:**
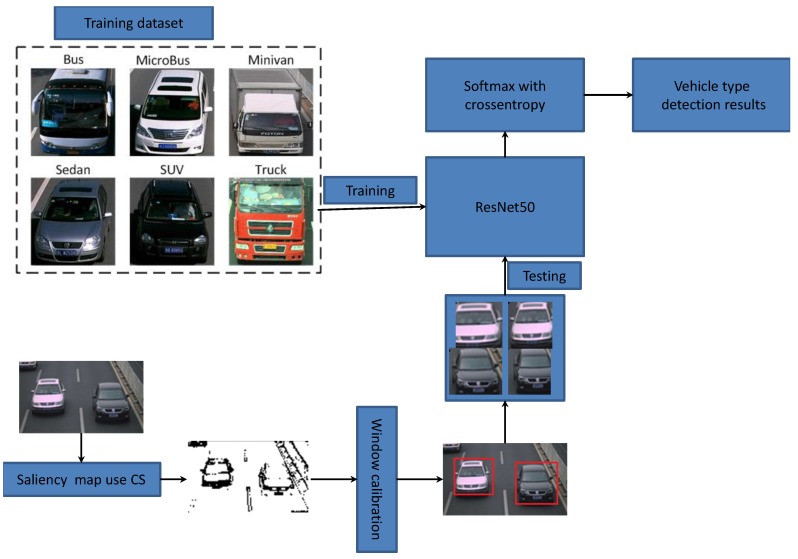
Diagram of vehicle-type detection based on CS and deep learning.

The process of vehicle-type detection is illustrated in Figure 5 [32]. In this study, we employed actual traffic-surveillance images from our laboratory and vehicles from the Internet as the training dataset, which contains sufficient samples of buses, sedans, and trucks. We trained the famous ResNet50 model using these massive images and then classified the testing images using Softmax with cross entropy.

## 5. Experimental Results

### 5.1. Saliency Map and Window-Calibration Results

**Figure 6:**
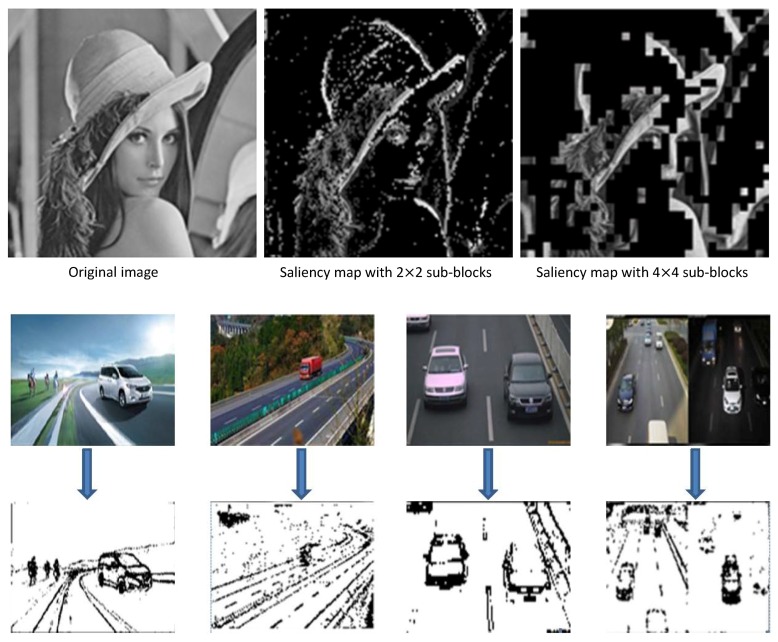
Saliency map results.

As shown in Figure 6, we tested using some images from the Internet to obtain their saliency maps with different block sizes. The test results indicate that whether the size of the image block is two or 16, saliency detection in on our method is very close to the effect achieved using an ideal saliency map. In this method, saliency analysis of the measurement domain is used to analyze the saliency of each column in the measurement value matrix and each sub-block in the original image through a simple threshold judgment after transformation. This greatly saves decoding time and improves efficiency.

The reason that we analyzed the saliency in the measurement domain rather than in the pixel domain is not just a matter of the measurement domain being a simple measurement of the pixel domain; there is also remarkable compression and reduced dimensionality in the measurement process. This significantly reduces the number of matrix elements that we need to process and further saves the time spent on image preprocessing. At the same time, CS is based on image segmentation, which can be later applied to the target window calibration. The calibrated window is rectangular, so the rectangular block is undoubtedly more convenient. This also facilitates the next target recognition.

Another advantage of saliency recognition based on CS over traditional saliency maps generated in the pixel domain is that the size of the blocks can be controlled. Traditional saliency-map generation is based on each pixel. We can change the size of the salient region in the saliency map by adjusting the threshold and filter parameters. However, the generated salient image still comprises continuous changes in pixels. The saliency decision based on block CS can be adjusted according to the size and complexity of the image. Generally, the larger the block, the lower the algorithm complexity. If the original image has a complex background or if the size of the image is small, we can reduce the size of the blocks and perform a detailed saliency analysis of the complex background. If the original image itself is relatively simple or the size of the image is large, we can use large sub-blocks to reduce the consumed time while satisfying the requirements for saliency analysis.

**Figure 7:**
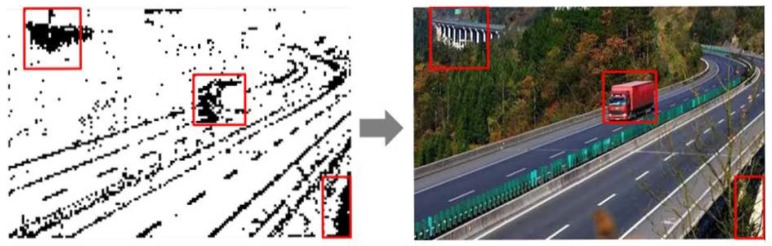
Window-calibration result based on the saliency map.

After obtaining the saliency map of an image, window calibration can be easily accomplished. As illustrated in Figure 7, we could find densely marked blocks and then uniquely match them to the area to be detected in the original image. The saliency analysis described in this paper remarkably reduces the background factors and removes a large number of redundant areas before window scanning. Just based on the proportion of black blocks in the window, we can quickly and easily select the suspected regions in the image.

Our method transforms the original image into an image with salient blocks whose colors are set to be merely black or white. Thus, the selection of windows can be effectively determined by simple area statistics. Therefore, the time complexity of our method is far less than that of the selective search algorithm in the overall window calibration.

### 5.2. Vehicle-Classification Results

The experimental environment used for this work was as follows: CPU Intel core i7-4790 3.6 GHz; memory 16 GB; GPU NVIDIA Quadro K2200, which contains 640 CUDA computing core units and 4 GB graphics memory. The simulation software was Matlab R2017a, and the program was written mainly based on the CNN ResNet50 for Matlab.

In this study, the original image data of the training samples were provided by our laboratory’s intelligent traffic big data project. We also captured pictures of different types of vehicles on the network. The original data samples were processed according to the experimental requirements and then used for training. At last, we chose all kinds of scenes to detect images of all kinds of vehicles and achieve a good accuracy for the target detection of the three vehicles types: sedans, buses, and trucks.

We compared the accuracy of our method with those in References [10,26,33,34]. For convenience, we refer to their methods as CS-CNN, raAdaBoost, and Haar+Cascade. Here, we employed public databases MIT CBCL and Caltech, as shown in Table 2.

Table 3 compares the average accuracy achieved with our proposed method and the accuracy of three existing methods. Curve precision, recall, and area average accuracy Average Precision (AP) formed by the curve are generally used as accuracy evaluation indicators in the field of target detection.

**Figure 8:**
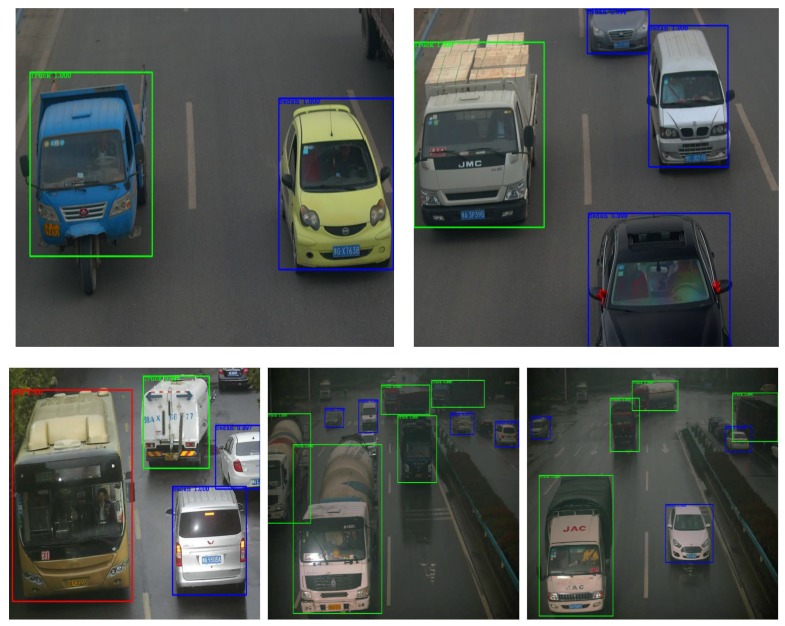
Effect of vehicle-type detection.

As demonstrated in Figure 8, we used actual traffic-monitoring images as the test input images. The three different types of vehicles, namely, sedans, buses, and trucks, were detected with high accuracy using the existing methods. Nevertheless, according to the result analysis, our proposed method achieves even greater accuracy.

## 6. Conclusions

In this paper, we proposed an efficient vehicle-type detection and recognition method that was generated by combining saliency mapping based on CS theory and the application of a CNN. In this method, an image is divided into blocks of the same size. Using CS theory, each block is projected into the measurement domain to acquire its measurements. By analyzing the sparse features of the measurements, the saliency map is extracted from the measurement domain. The use of the measurement domain not only obtains a simple measurement of the pixel domain, but also achieves remarkable compression and dimensionality reduction in the measurement process. This significantly reduces the number of matrix elements that need to be processed, and further saves time spent on image preprocessing. Furthermore, the calibrated window is rectangular, so the rectangular blocks in the CS process are undoubtedly more convenient. Based on the saliency map, the salient regions and the suspected targets in an image can easily be found; thus, window calibration can be efficiently completed. In addition, we adopted ResNet50 for the classification process. The main advantage of ResNet is that it can use a deeper network to solve the problem of increased training errors with increased network layers. Our proposal reduces the requirements for high computation and a large amount of training data. The experimental results demonstrate that compared with some traditional machine learning methods, our method is able to speed up the window calibrating stages of CNN-based image classification. Furthermore, our method achieves higher accuracy when the amount of training data is restricted. In summary, our proposal has better overall performance in vehicle-type detection than some of the traditional methods. It has very broad prospects for practical applications in vehicular networks.

## Figures and Tables

**Table 1:** Structure of ResNet.

Layer Name	Output Size	18-Layer	34-Layer	50-Layer
cov1	112×112	7×7, 64, stride 2		
cov2_x	56×56	3×3 max pool, stride 2		
		$\left[\begin{array}{cc} 3\times 3, & 64\\ 3\times 3, & 64 \end{array}\right]\times 2$	$\left[\begin{array}{cc} 3\times 3, & 64\\ 3\times 3, & 64 \end{array}\right]\times 3$	$\left[\begin{array}{cc} 1\times 1, & 64\\ 3\times 3, & 64\\ 1\times 1, & 256 \end{array}\right]\times 3$
cov3_x	28×28	$\left[\begin{array}{cc} 3\times 3, & 128\\ 3\times 3, & 128 \end{array}\right]\times 2$	$\left[\begin{array}{cc} 3\times 3, & 128\\ 3\times 3, & 128 \end{array}\right]\times 4$	$\left[\begin{array}{cc} 1\times 1, & 128\\ 3\times 3, & 128\\ 1\times 1, & 512 \end{array}\right]\times 4$
cov4_x	14×14	$\left[\begin{array}{cc} 3\times 3, & 256\\ 3\times 3, & 256 \end{array}\right]\times 2$	$\left[\begin{array}{cc} 3\times 3, & 256\\ 3\times 3, & 256 \end{array}\right]\times 6$	$\left[\begin{array}{cc} 1\times 1, & 256\\ 3\times 3, & 256\\ 1\times 1, & 1028 \end{array}\right]\times 6$
cov5_x	7×7	$\left[\begin{array}{cc} 3\times 3, & 512\\ 3\times 3, & 512 \end{array}\right]\times 2$	$\left[\begin{array}{cc} 3\times 3, & 512\\ 3\times 3, & 512 \end{array}\right]\times 3$	$\left[\begin{array}{cc} 1\times 1, & 512\\ 3\times 3, & 512\\ 1\times 1, & 2048 \end{array}\right]\times 3$
	1×1	average pool, 1000-d fc, softmax		
FLOPs		$1.8\times 10^{9}$	$3.6\times 10^{9}$	$3.8\times 10^{9}$

**Table 2:** Used public databases.

	MIT CBCL	Caltech Database
Number of vehicle images	439	652
Image size	128 × 128 pixels	240 × 360 pixels

**Table 3:** Comparison between the accuracy of our proposed method and other methods.

Methods	MIT CBCL	Caltech Database
Haar+Cascade	0.9338	0.9238
raAdaBoost	0.9355	0.9302
CS-CNN	0.9371	0.9427
PROPOSED	0.9412	0.9504

## Abbreviations

The following abbreviations are used in this manuscript:

CS	Compressed Sensing
CNN	Convolutional Neural Network
